# *laminin alpha 1 *gene is essential for normal lens development in zebrafish

**DOI:** 10.1186/1471-213X-6-13

**Published:** 2006-03-07

**Authors:** Natalya S Zinkevich, Dmitry V Bosenko, Brian A Link, Elena V Semina

**Affiliations:** 1 Department of Pediatrics, Medical College of Wisconsin, Milwaukee, WI 53226, USA; 2 Departments of Cell Biology, Neurobiology and Anatomy, Medical College of Wisconsin, Milwaukee, WI 53226, USA; 3 Departments of Human and Molecular Genetics Center, Medical College of Wisconsin, Milwaukee, WI 53226, USA

## Abstract

**Background:**

Laminins represent major components of basement membranes and play various roles in embryonic and adult tissues. The functional laminin molecule consists of three chains, alpha, beta and gamma, encoded by separate genes. There are twelve different laminin genes identified in mammals to date that are highly homologous in their sequence but different in their tissue distribution. The *laminin alpha -1 *gene was shown to have the most restricted expression pattern with strong expression in ocular structures, particularly in the developing and mature lens.

**Results:**

We identified the zebrafish *lama1 *gene encoding a 3075-amino acid protein (lama1) that possesses strong identity with the human LAMA1. Zebrafish *lama1 *transcripts were detected at all stages of embryo development with the highest levels of expression in the developing lens, somites, nervous and urogenital systems. Translation of the *lama1 *gene was inhibited using two non-overlapping morpholino oligomers that were complementary to sequences surrounding translation initiation. Morphant embryos exhibited an arrest in lens development and abnormalities in the body axis length and curvature.

**Conclusion:**

These results underline the importance of the *laminin alpha 1 *for normal ocular development and provide a basis for further analysis of its developmental roles.

## Background

Basement membranes play an important role in tissue development and maintenance including mechanical stability, formation of barriers between different cell types and promotion of cell adhesion, migration, growth and differentiation. Laminins are large glycoprotein heterotrimers that are found as major components of basement membranes in almost every animal tissue. To date, five *alpha*, four *beta*, and three *gamma *precursors have been identified that can combine to form fifteen laminin isoforms with different tissue distribution [1-3]. Mutations in laminin genes have been identified in several human disorders: muscular dystrophy (*LAMA2*; [4]), epidermolysis bullosa and Laryngo-onycho-cutaneous syndrome (*LAMA3 *[5,6]; *LAMB3 *[7]; *LAMC2 *[8], and microcoria-congenital nephrosis syndrome (*LAMB2 *[9]).

Laminin-1, which is composed of alpha-1, beta-1 and gamma-1 chains, was first described by Timpl and co-authors in 1979 [10]. Laminin-1 shows restricted expression that is largely limited to epithelial basement membranes. Laminin-1 is detected in most embryonic tissues during early morphogenesis and remains present as a major epithelial laminin in some adult tissues [2,11-13]. Mice that are deficient in any chain that composes laminin-1 (α1β1γ1) die during the early postimplantation period with the *Lama1*-/- phenotype being the mildest of the three genes deleted [14,15]. This finding could be explained by the fact that β1 and γ1 proteins participate in multiple heterotrimers and therefore have broader functions than α1 chain that is restricted to two laminins. Other animal models of laminin-1 deficiency include zebrafish *grumpy *(β1) and *sleepy *(γ1) mutants that were identified in a genome-wide chemical mutagenesis screen [16,17] and *lamb1 *and *lamc1 *(several alleles) mutants produced by retrovirus-mediated insertional mutagenesis [18,19]. The zebrafish *laminin β1 *and *γ1 *mutants display shortened body axes due to a failure of notochord differentiation as well as complex ocular defects ([16-19]; also see below). To date, there are no distinct human phenotypes associated with laminin-1 mutations although some studies suggested a potential involvement of *LAMB1 *in a neonatal cutis laxa with a Marfan phenotype [20] and *LAMC1 *in a junctional epidermolysis bullosa inversa [21].

The *laminin alpha-1 *gene shows a tissue-restricted expression pattern and is considered to be the most specific of the classical laminins. Expression of *lama1 *is detected in the nervous and urogenital systems, pre-somitic mesoderm, some brain blood vessels and in the embryonic and mature lens ([12,13,15], and [22]). The important role of laminins/extracellular matrix/basement membranes in eye development and in an adult ocular function has been discussed in several reports [23-26] but the specific roles of different laminin subunits are only beginning to be elucidated.

Besides laminin-1 (α1β1γ1), laminin alpha-1 participates in one additional trimer, laminin-3 (α1β2γ1) [11,27]. Interestingly, except for lama1, all other components of either laminin-1 or -3 were found to be involved in ocular developmental phenotypes. Human *LAMB2 *mutations result in a complex phenotype that includes such ocular manifestations as microcoria, lenticonus, Rieger anomaly, glaucoma, cataracts and microphthalmia [9]. Mutations in *laminin β1 *and *γ1 *genes result in multiple eye anomalies in zebrafish: retinal blowout (expulsion of retinal cells through the RPE into the adjacent forebrain) [19], disorganized optic nerves [28], some retinal lamination defects [18,19] and lens hypoplasia, lens capsule rupture and corneal defects [19]. As laminin alpha 1 contributes to both laminins, *lama1 *mutations are likely to result in similar eye defects and may even cause more severe and/or complex ocular phenotypes due to the cumulative effect of laminin-1 and -3 deficiencies.

Zebrafish represents a valuable vertebrate model to study developmental processes. In this report, we present identification and characterization of the zebrafish *laminin alpha *1 gene including its sequence, expression pattern, and loss-of-function phenotype.

## Results

### Cloning of zebrafish lama1 gene

In order to identify the zebrafish *laminin alpha 1 *gene, we first performed a search for homologous sequences using the known human and mouse *laminin α1 *sequences, zebrafish genomic database (Zv3) [29] and BLAST engine. This approach identified ten sequences homologous to the human *LAMA1 *gene with the most upstream sequence corresponding to exon 4 and the most downstream one- to exon 51 of the human *LAMA1 *gene (the entire human gene contains sixty-three exons (GenBank accession number NM_005559)). The identified sequences were used to design specific oligonucleotides that were then utilized in RT-PCR reactions using RNA isolated from 48-hpf *Danio rerio *embryos; the resultant PCR products were separated by electrophoresis, cloned into a plasmid vector and subjected to DNA sequencing. To identify the full-length *lama1 *transcript, we performed 5'- and 3' RACE reactions and determined sequences for the corresponding products of these reactions. The obtained sequences were arranged into a contiguous assembly and analyzed using Vector NTI™ sequence analysis software.

**Figure 1:**
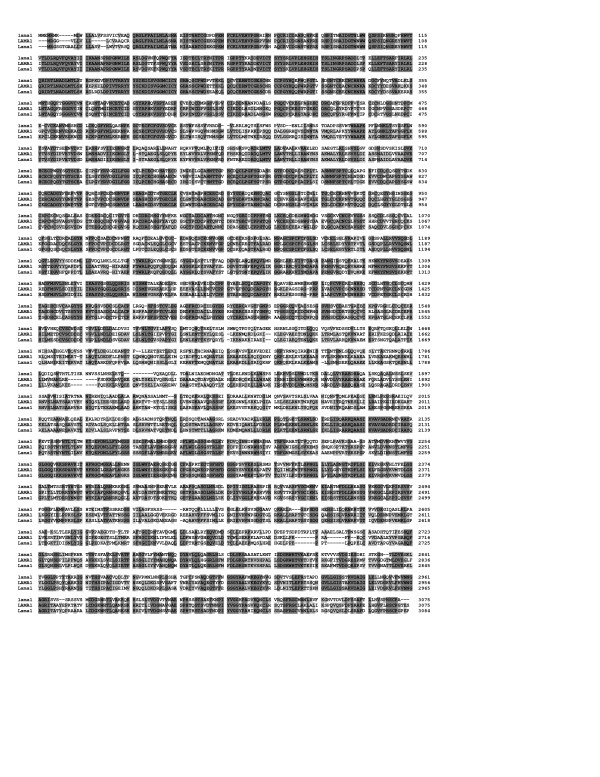
**Alignment of laminin alpha 1 proteins. **The amino acid residues that are identical in all three or in any two of the proteins (lama1 (zebrafish), LAMA1 (human), Lama1 (mouse)) are highlighted in dark or light grey, respectively.

The *lama1 *cDNA contig comprised 9581-bp and contained a 9225-bp open reading frame that was predicted to encode 3075 amino acid protein (Figure 1), 128-bp of 5'UTR and 228-bp of 3'UTR sequence (GenBank number DQ131910). Detailed analysis of the 5' sequence identified five initiator codon trinucleotides (ATG) in the 24-bp region spanning nucleotides 128-152. Among these potential translational start sites, the second ATG appears to have the most favorable surrounding sequence GCGATG**ATG**G with four nucleotides (underlined) being consistent with the Kozak's consensus sequence identified for vertebrate genomes [30]. As the translational site "context" sequence is not exclusive at any positions and some sites were found to be occupied by non-conserved nucleotides in all five sequences, we selected the most upstream ATG codon as a translational start site for the lama1 protein.

**Figure 2:**
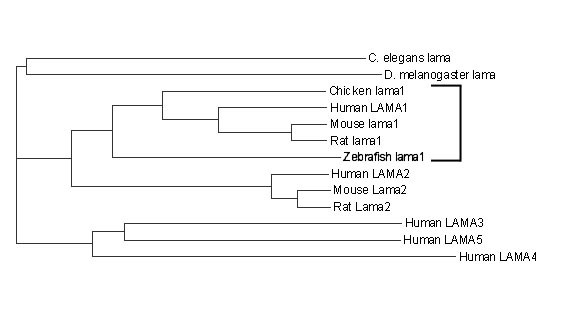
**Phylogenetic tree analysis of the laminin alpha proteins. **The zebrafish protein demonstrates close relationship with laminin alpha 1 proteins from other species (laminin alpha 1 cluster is indicated by bracket).

The overall sequence demonstrated strong homology with laminin genes/proteins of the *alpha *family that were shown to be highly homologous to each other. In order to position the novel zebrafish gene within the *alpha *laminin family, we performed a phylogenetic tree calculation using the corresponding module of Vector NTI™ suite. This algorithm is based on a sequence distance method and utilizes the Neighbor Joining formula of Saitou and Nei [31]. This analysis demonstrated grouping of the novel transcript with the *laminin alpha 1 *sequences from other species (Figure 2). The comparison of human, mouse and zebrafish laminin α1 amino acid sequences showed high identity level throughout the entire protein (Figure 1). Based on BLAST analysis results, the overall zebrafish lama1 sequence demonstrated ~51% identity with human, mouse and chicken laminin α1, and ~42% identity- with human and mouse laminin α2 proteins. The laminin alpha 1 contains several conserved domains: short signal peptide (amino acids 1 through 17), N-terminal region (a.a. 18-269), seventeen laminin EGF-like domains and two laminin IV type A1 domains (a.a. 270- 1555), and five laminin G-like domains (a.a. 2305-3070) (regions are indicated according to the human LAMA1 protein, GenBank number P25391). The N-terminal domain demonstrated the highest level of conservation (89% identity with human or mouse sequence) while the identity level in other domains varied from ~30% to 75%. The central region of the laminin alpha 1 protein encompassing amino acids 1555-2085 (this region participates in the coil-coil domain formed by three chains α1,β1, and γ1) demonstrated the lowest level of conservation at ~30% (Figure 1). The zebrafish *lama1 *nucleotide and protein sequences were submitted to GenBank with accession number DQ131910.

### Identification of genomic structure of the lama1 gene

Genomic sequences of the *lama1 *gene were identified either by sequence similarity search using cDNA sequences, BLAST engine and public databases (Zebrafish Whole Genome Sequencing database; 32) or by direct sequencing of products generated by long-range PCR using exonic primers and genomic DNA. The gene was found to consist of sixty-three exons ranging from 87 to 378 bp in length. Overall, the genomic structure of the zebrafish *laminin alpha 1 *gene corresponded well with the human *LAMA1*; all the donor and acceptor splicing sites contained characteristic consensus sequences conserved in vertebrates (Table 1).

### Embryonic expression of zebrafish lama1

**Figure 3:**
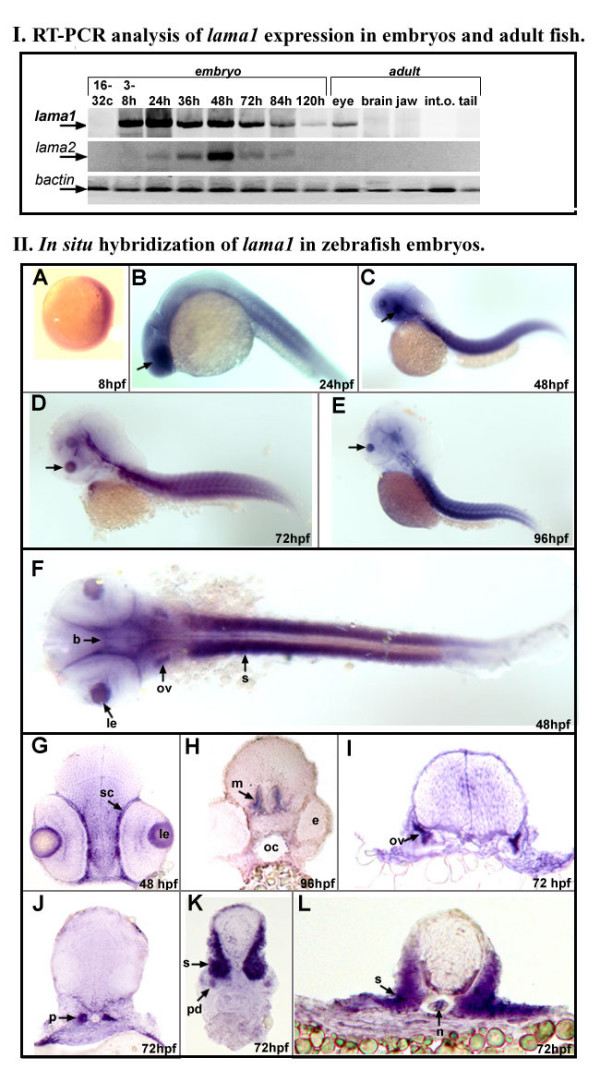
**Expression of zebrafish laminin alpha 1 gene**. **I. **RT-PCR analysis of lama1 expression in embryos and adult fish. RT-PCR results for *lama1, lama2 *and control *bactin *transcripts are presented as indicated. Embryonic (16-32 cells to 120-hpf) or adult (1 year old) cDNA samples employed in reactions are indicated at the top: lane 1- 16-32 cells, 2- 3-8 hpf, 3- 24 hpf, 4- 36 hpf, 5- 48 hpf, 6- 72 hpf, 7- 84 hpf, 8- 120 hpf embryos; for adult tissues- lane 9 contains products obtained with adult eye cDNA, 10- brain, 11- jaws, 12- internal organs and 13- tail. **II**. *In situ *hybridization of antisense *lama1 *riboprobe in zebrafish embryos. **A-F**: 8-96 hpf whole zebrafish embryos that were hybridized with *lama1 *DIG-labeled antisense riboprobe. **G-L**: Transverse sections of 48-96 hpf zebrafish embryos at the level of the eye (**G**), brain (**H**), otic vesicle (**I**), developing kidney (**J**), and trunk (**K**, **L**). Embryonic stages are indicated at the bottom of the picture. At 8-hpf, expression of the *lama1 *gene was detected in all embryonic tissues; by 24-hpf, higher levels of transcript were evident in the developing lens (arrows in B-E; **le **in F and G) and sclera (**sc**) of the eye, brain (**b**), somites (**s**), and otic vesicle (**ov**), pronephros (**p**) and pronephric duct (**pd**), notochord (**n**). **e**- eye, **m**-midbrain.

Embryonic expression of the zebrafish *lama1 *gene was studied by RT-PCR and *in situ *hybridization. Embryos ranging from the 16-32-cell stage to 120-hpf, as well as different adult tissues were examined for the presence of the *lama1 *transcript. Expression of *lama1 *was strong during embryonic development and depleted in most adult tissues, which is consistent with the previously reported data ([12,13], and [22]). First *lama1 *transcripts were detected in 3-8 hpf embryos (encompasses embryos at 1k-cell stage of blastula to 75%-epiboly stage of gastrula) and expression continued at later embryonic and larval stages of development (Figure 3). In adult fish, expression was observed in the eye.

We also tested expression of an additional laminin transcript, *lama2*-like. The Lama1 and Lama2 proteins are highly homologous to each other and were shown to be functionally redundant [33,34]. The *Lama1&2 *genes are expressed in separate as well as overlapping domains during development including ocular tissues [35-39]. The 1776-bp *lama2*-like sequence was identified from GenBank (Accession Number XM_693031) and demonstrated 61% identity with the human LAMA2 at amino acid level. Expression of zebrafish *lama2-*like gene was tested by RT-PCR with gene-specific primers. Based on RT-PCR results, *lama2 *expression is detectable starting from 24-hpf embryos (pharyngula) to 84-hpf larvae and was not found in adult tissues. The *lama2 *and other, not yet identified, zebrafish *alpha *transcripts are likely to be able to substitute for *laminin alpha 1 *and each other during embryonic development. Identification and characterization of these genes is necessary to better understand multiple roles of different laminin isoforms during development.

Whole mount *in situ *hybridization was performed using embryos at 24-, 48-, 72-, and 96-hpf and a 590-bp antisense riboprobe that comprised *lama1 *sequence corresponding to nucleotide positions 287-876 (GenBank accession number DQ131910). Expression of *lama1 *was detected in the developing lens, sclera, midbrain, somites, urogenital system and notochord (Figure 3), which is consistent with the *Lama1 *gene expression in other species.

### Morpholino-mediated knockdown of zebrafish laminin alpha 1 expression

To examine the functions of laminin alpha 1 during embryonic development, we injected *lama1*-specific and control oligonucleotides into 1-2 cell stage embryos. The *lama1 *morpholinos were designed to hybridize to the 5' sequence of the *laminin alpha 1 *mRNA near the initiation codon (position 1): MO1 oligomer corresponds to sequence from nucleotide -65 to -39 while MO2 morpholino matches sequence between nucleotides at positions -3 and +22.

**Figure 4:**
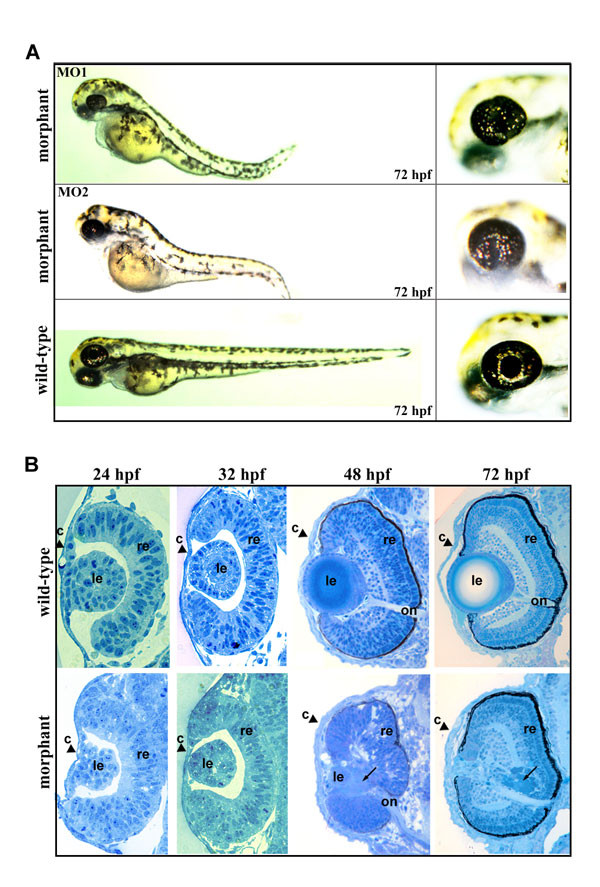
**lama 1 knockdown phenotype in zebrafish. A**, an overall view of *lama1*-morphants obtained with MO1- (top) or MO2- oligomers (middle), and control (bottom) embryos at 72-hpf. Enlarged image of a head is provided on the right. Defects in body length, axis curvature and eye structure (irregular pupil and a lack of lens) are easily detectable in *lama1 *morphants. **B**, transverse sections at the eye level of control (top row) and *lama1*-morphant (bottom row) embryos at 24-, 32-, 48- and 72-hpf are presented. An obvious lens degeneration is first notable in 48-hpf morphant eyes. At 72-hpf, small eyes with missing lens and thickened cornea were observed in the morpholino-injected embryos. retina (**r**), optic nerve (**on**), lens (**le**) and cornea (**c**) are shown. Black arrows in 48- and 72-hpf eyes indicate to the products of lens degeneration.

The morphological phenotype in the morpholino-injected embryos was first detected in 36-hpf embryos and became highly evident by 72-hpf. The morphants exhibited a shortened body, an abnormal body axis curvature, and malformed eyes that often lacked lenses and had misshapen pupils (Figure 4). The percentage of morphants exhibiting the phenotype positively correlated with the concentration of the injected morpholinos and ranged from 30% (0.25 mM; total number = 165) to 60% for MO1 oligomer (0.5 mM; total number = 658) or 50% for MO2 (0.5 mM; total number 650). The rate of early lethality by 24-hpf ranged from 7% (for C = 0.25 mM) to 23% for MO1 (for C = 0.5 mM) or 31% for MO2 oligomer (for C = 0.5 mM). In control experiments, zebrafish embryos that were injected with control morpholinos as well as uninjected larvae were examined for morphological phenotypes. Both groups demonstrated phenotypes indistinguishable from the wild-type fish with a lethality rate of ~10% level for un-injected larvae and embryos that were injected at C = 0.25 mM; embryos that were injected at C = 0.5 mM demonstrated 21% lethality.

To determine ocular defects in the *lama1*-morphants, we compared the histology of the *lama1 *morphant eyes to wild-type in embryos ranging from 18-hpf to 72-hpf (18-hpf- data not shown; sections of 24- to 72-hpf embryos are presented at Figure 4). Since morphant fish produced by injections with either MO1 or MO2 oligomers demonstrated similar phenotypes based on visual examination, only MO1- injected morphants have been used for further histological analysis. For 48-hpf and 72-hpf stages, embryos with abnormal phenotype were selected; while for the 18-, 24- and 32- hpf time points, twelve MO1-oligomer- injected living embryos were collected before the phenotype is evident and eight (65%) of these animals were expected to be morphants (this estimate was based on the fact that 65% of embryos from the same group that were raised till 72-hpf demonstrated an ocular morphant phenotype). The stages of the establishment of the lens placode, lens delamination from the ectoderm and formation of the lens vesicle appear to be grossly normal in *lama-1 *deficient embryos. The cells of the lens vesicle seem to be slightly disorganized in about 20% of 24-32-hpf embryos (Figure 4), which is below the estimated frequency of morphant embryos in this sample. Therefore, we concluded that formation of the lens vesicle appears to be mostly unaffected in *lama-1 *morphants. In the 48-hpf morphant eyes, a small lens vesicle remnant was found and it was surrounded by degenerating lens tissue. In the 72-hpf morphants, the lens was absent and lenticular bladder cells seem to form a deposit in subretinal space in some embryos (Figure 4). In normal 72-hpf animals, the following main ocular tissues can easily be detected: 1) laminated retina containing the photoreceptor, inner nuclear and ganglion cell layers; 2) lens that consists of a single layer of epithelial cells and mainly differentiated lens fibers and 3) cornea with three easily observed layers- surface epithelium, a thin lamellar stroma that is contiguous with sclera and flattened endothelial cells [40]. In addition to lens degradation, the eye sections obtained from 72-hpf *lama1 *morphant embryos revealed different degrees of thickened cornea and reduced eye size (Figure 4). At the same time, the retina and the optic nerve are present and appear to be grossly normal in the *lama1*-knockdown fish.

## Discussion

In this paper, we report identification of the zebrafish *laminin alpha 1 *gene and analysis of its role during embryonic development by means of expression and knockdown studies. Our data indicate a strong conservation of *lama1 *function during vertebrate development as both the predicted protein sequence and expression pattern of this gene were found to be highly conserved between zebrafish and other species. The highest level of conservation was identified in the N-terminal domain of lama1 followed by the laminin globular (G-like) domains. The N-terminal domain was shown to be involved in laminin polymerization in vitro [41,42], and binding to integrins α1β1 and α1β2 [43]. The five laminin globular domains located in the C-terminus represent the main cell-adhesive sites and bind the major laminin receptor integrin α6β1 as well as α6β4 and α7β1 [44], extracellular heparan proteoglycan perlecan, dystroglycan, sulfatides and heparin [45]; mice lacking the alpha 1 chain LG4-5 module were reported to die at E6.5 with failure of epiblast differentiation [46]. Preservation of the N-terminal and C-terminal domain sequences throughout vertebrate evolution suggests that the interactions mediated by these regions are of particular importance.

The zebrafish *lama1 *gene was found to be strongly expressed during embryonic development. The following high-expression sites were identified in the developing embryo: lens, brain, somites, urogenital system, and notochord. This pattern is consistent with distribution of *Lama1 *transcripts in other species ([12,13,22,41,47], and [48]) and suggests a high degree of conservation in *laminin alpha 1 *function during embryonic development in vertebrates. The evolutionarily conserved expression of *lama1 *is likely to be governed by a network of specific regulatory elements maintained in phylogenetically divergent species. Identification of *cis*-regulatory regions and *trans*-acting factors that direct the specific *lama1 *expression pattern will provide important insight into mechanisms of embryonic development and ocular tissue maintenance.

Knockdown of the laminin alpha 1 expression in zebrafish resulted in a distinct phenotype characterized by anomalies in eye development as well as body axis length and curvature. This condition is different from the phenotype reported in *Lama1-/- *knockout mice [15]. The *Lama-/- *null mice die prenatally around day 7 post coitus (pc) while the embryos that are deficient in either β1 or γ1 laminin chains do not survive past day 5.5 pc, which is the blastula stages in mice [15]. The laminin-1 is first detected around the 16-cell stage in mice and present in the two basement membranes formed before gastrulation. Mammalian embryos deficient in any component of laminin-1 (α1β1γ1) survive implantation but die before gastrulation indicating to the critical role of laminin-1 in this process [15,49]. In zebrafish, implantation does not occur as embryogenesis occurs *ex utero*. Gastrulation does not seem to require laminin-1 in zebrafish as both *lamb1*- and *lamc1*-mutants undergo normal germ-layer patterning and gastrulation movements [17]. This phenomenon may be explained by a compensation from another laminin or differences in mechanisms between fish and mammals. Identification and studies of all other zebrafish laminin isoforms are necessary to clarify this issue.

The ocular phenotype in zebrafish embryos deficient in *laminin alpha 1 *is characterized by slightly smaller eyes with visible anomalies in lens and cornea development. A primary defect may be the lens degeneration due to developmental arrest that causes collapse of the surrounding ocular tissue (reduction in eye size) and abnormal patterning of the anterior segment structures (cornea defects). Similar associations between lens defects, small eye and malformed anterior chamber have been previously reported [50-58]. At the same time, the visible corneal defects may indicate a discrete function of *lama1 *in the development of the anterior segment structures.

The severe ocular phenotype observed in the *lama1*-deficient fish embryos reveals a new role for this molecule during vertebrate embryonic development. The observed *lama1*- knockdown phenotype is consistent with the ocular abnormalities associated with other laminins such as *lamb1 *and *lamc1 *in zebrafish and *LAMB2 *in humans ([9,18,19], and [28]). Interestingly, all these proteins are involved in the only two trimers that *laminin alpha 1 *was found to be a part of: laminin-1 (α1β1γ1) and laminin-3 (α1β2γ1). The Lamb1 and Lamc1 proteins are widely expressed in different species and can associate with any alpha laminin. Mutations in *lamb1 *and *lamc1 *in zebrafish result in complex phenotypes that include lens hypoplasia, lens capsule rupture and corneal defects [19]. The laminin-3 was originally identified in human placenta [27] but *Lamb2 *mRNA has also been detected in lens, corneal, pigment epithelial and hyaloid cells during development [9,59-61]. Clear evidence of an important role of beta-2 and its complexes in human ocular development was provided by the discovery of *LAMB2 *mutations in human patients affected with complex ocular phenotypes that include lens, iris, corneal, retinal and overall eye-size defects [9]. The exact role(s) of laminin-1 and -3 during vertebrate eye development require further investigation. Identification and functional analysis of zebrafish *lamb2 *may provide an important insight into this issue.

There are several ocular phenotypes that involve lens degeneration including *aphakia *[62-64], *dysgenetic lens *[65-67], *lens aplasia *[68,69] in mice. Genes responsible for the *aphakia *and *dysgenetic lens *phenotypes have been identified as transcription factors, *Pitx3 *and *Foxe3*; both genes were also shown to be involved in human ocular disorders involving abnormal lens, iris and corneal development [54,55] and zebrafish *pitx3*-morphants displayed lens degeneration similar to mammals [70]. Defects in the basement membrane and/or extracellular matrix were reported in *aphakia *and *lens aplasia *mutants [69,71] indicating a possible connection with the laminin and/or other extracellular matrix molecule pathway(s) that needs to be further investigated.

The lens is surrounded by the lens capsule that represents a thick basement membrane that includes laminins, collagen IV, heparan sulfate proteoglycans (perlecan), nidogen and fibronectin. The other components, such as type XV and type XVIII collagen, agrin, fibulins and growth factors, may be present at some stages as well. The importance of the extracellular matrix/basement membrane for lens development was proposed based on the distinctive spatio-temporal expression patterns of different extracellular matrix proteins during lens development [23,72-74], changes in the distribution of the extracellular matrix proteins during normal and aberrant lens development [23,69,71,75] as well as human and animal phenotypes associated with mutations in ECM component genes that, in addition to the above discussed, include perlecan [76] and collagen XVIII [77].

Based on the phenotype observed in zebrafish, mutations in the laminin-1 components are likely to contribute to human disorders of the lens (cataracts) and/or anterior segment development (glaucoma). Also, because the lens plays an important role in normal ocular growth, defects in lens development may also play a role in another common ocular disease- myopia [78]. This possibility is further supported by the fact that the messenger RNA for *lama1 *was detected in the developing sclera in addition to the lens in zebrafish; sclera cell development has been shown to be important for the normal eye growth in several studies [79-82]. In humans, *LAMA1 *maps to the 18p11.31 region that contains a gene for high-grade myopia (MYP2; [83]). The affected individuals were characterized by an average spherical component refractive error of -9.48 diopters and an average age at diagnosis of myopia of 6.8 years; no clinical evidence of connective tissue abnormalities has been noted ([83]). Because of the essential role of laminin-1 in governing early events in mammalian development, human laminin-1 mutations in ocular phenotypes, if any, are most likely to be detected in a heterozygous state and/or to be specific to the particular interactions involved in eye development and maintenance. The expression pattern of *LAMA1 *in human ocular tissues needs to be determined and the potential contribution of this gene in ocular disease should be examined.

## Conclusion

The *laminin alpha 1 *gene was found to play an important role in ocular development in zebrafish. Given that *Lama1 *was shown to be expressed in eye tissues in mammals as well, this gene is likely to have a similar role in these higher species. Additional studies into the specific role(s) of laminin-1 and laminin-3 during eye development are necessary. The findings can then be correlated with specific human phenotypes to identify mutations that may impair different regions of this complex molecule.

## Methods

### Animals

Zebrafish (*Danio rerio*) were raised and maintained on a 14-hour light/10-hour dark cycle. The embryos were obtained by natural spawning and raised at 28.5°C. The developmental stage was determined by time (hours post fertilization (hpf)) and by morphological criteria [84]. All experiments were conducted in accordance with the guidelines set forth by the animal care and use committees at the Medical College of Wisconsin.

### Cloning of lama1: RT-PCR, RACE, long-range PCR, cloning and sequencing

PCR products were generated using specific oligos, *PfuUltra *high-fidelity DNA polymerase (Stratagene, La Jolla, CA) and standard conditions described elsewhere [70]. The PCR products were separated by electrophoresis in 1% agarose gel, cloned into a pCRII-TOPO vector (Invitrogen, Carlsbad, CA) and subjected to DNA sequencing using the ABI PRISM 373 DNA Sequencer. The 5'- and 3' RACE (Rapid Amplification of CDNA Ends) was performed using BD SMART™ RACE cDNA Amplification Kit (Clontech, Mountain View, CA) and the following oligonucleotides: 5'- ACCACAGGTTGGTTCCATCGATG-3' for the 5'RACE and 5'-GCGGACCACACACAGACCATCCC-3' – for the 3' portion of the transcript. The overlapping *lama1 *sequences were analyzed and arranged into contig using Vector NTI™ sequence analysis software. The long-range PCR was performed using TripleMaster™ PCR System (Eppendorf, Hamburg, Germany) and conditions suggested by the manufacturer.

### Expression analysis: RT-PCR and tissue in situ mRNA hybridization

For the RT-PCR reaction: the *lama1 *specific oligonucleotides complimentary to sequences at positions 3031-3050, 5'-TGTCTGCGTCATGTGATGAG-3', forward primer, and positions 8440-8421, 5'-TCGCCATGTAGAACAGAACG-3', reverse primer, were used to amplify 5406-bp *lama1 *products from cDNA extracted from 16-cells to 120-hpf embryos. The sequence predicted to represent zebrafish *lama2 *gene was identified from the database (GenBank number XM_693031) and the following primers were used to amplify 275-bp gene-specific product: forward, AAGCATCATGAACGGGATGG, and reverse, TGGAGTAGAAGGAGGTACAG. Control primers, 5'-GAGAAGATCTGGCATCACAC-3', forward and 5'-ATCAGGTAGTCTGTCAGGTC-3'- reverse primer, were used to amplify 324 -bp fragment of *beta-actin *gene. For the *lama1 in situ *hybridization, the following probe was prepared: a 590-bp fragment that comprised *lama1 *sequences corresponding to nucleotide positions 287-876 and 1475-3031 (GenBank accession number DQ131910) was subcloned into pCRII-TOPO plasmid (Invitrogen, Carlsbad, CA) and used as a template for making an antisense riboprobe. The digoxigenin-labeled antisense riboprobe was prepared using DIG RNA Labeling Kit (Roche Applied Science, Indianapolis, IN) and manufacturer protocols. Anti-DIG AP (1:2000) and NBT/BCIP substrate (Roche Applied Science, Indianapolis, IN) were used to detect the probes. Wild-type PTU-treated zebrafish embryos at 8- 96 hpf were fixed in 4% paraformaldehyde/PBS then washed in PBS and fixed in 100% MeOH. Then whole-mount in situ embryos were fixed in 4% paraformaldehyde/PBS and infiltrated with 2-h steps of 15% sucrose, 30% sucrose and 100% Tissue-Tek OCT (Miles Inc., Elkhart, IN). Fifteen to twenty embryos were oriented in freezing molds and stored at -20°C until sectioning. Ten-micrometer sections were cut on a cryostat and mounted on gelatin-coated glass slides.

### Morpholino oligomer injections and histology

The *lama1*-specific morpholino oligomers were designed using Gene Tools (Corvallis, OR) services and purchased from the company. Two oligomers were made to hybridize to the sequence in the 5' UTR of *lama1 *transcript: MO1, 5'- ATAAAGCTAAAGCTGTGCTGAAATC-3', and MO2, 5'- TCTTCATCCTCATCTCCATCATCGC-3'. Control oligomer: 5'-AAACAAACCTGAGGACAGATGGA-3'. The morpholinos were resuspended in water and injected into 1-2 cell stage embryos using Nanoject II injector (Drummond Scientific, Broomall, PA) or MM33 Mircomanipulator (Stoelting Co., Wood Dale, IL) as described elsewhere [85]. Approximately eight (MM33) or fifteen (Nanoject II system) nanoliters of oligomer mixture was injected into each 1-2 cell embryo. The embryos injected with *lama1- *or control morpholino oligos as well as un-injected embryos were allowed to develop at normal temperature (28.5°C) and examined for morphological phenotypes every 6-24 hours.

For the histological analysis of the 48-hpf and 72-hpf stages, morphant embryos that exhibited short body and abnormal eye phenotype were identified and collected. For the examination of the 18-hpf to 32-hpf embryos, twelve living embryos were collected for every stage following MO1-morpholino injection; fifty embryos from the same group were monitored till 72-hpf and 65% of these animals demonstrated mutant phenotype. Therefore a mixture of ~1/3- wild-type and 2/3- morphant embryo sections was expected to be present at 18-, 24- and 32-hpf slides. Histological specimens were processed as previously described [40]. In brief, embryos were fixed in primary fixative [2% paraformaldehyde, 2.5% glutaraldehyde, 3% sucrose, 0.06% phosphate buffer (pH 7.4)] at 4°C for 24 hours and then washed in 0.1 M phosphate-buffered saline (PBS), dehydrated through an ethanol series and propylene oxide and then infiltrated with EMbed-812/Araldyte resin mixture. The 1 μm- thin plastic sections were cut with a glass knife on a JB4 microtome. Sections were stained with 1% Toluidine Blue in 1% Borax buffer. Images were captured using a Nikon coolpix 995 digital color digital camera mounted on a Nikon E800 compound microscope with a 60X oil-emersion objective.

## List of abbreviations used

*lama1*- *laminin alpha 1*; RT-PCR- reverse transcription polymerase chain reaction; RACE- rapid amplification of cDNA ends; hpf- hours post fertilization.

## Authors' contributions

Natalia Zinkevich performed *in situ *hybridization analysis of *lama1 *expression and knockdown studies. Dmitry V. Bosenko carried out *lama1 *gene sequence identification and RT-PCR analysis. Brian A. Link participated in an experimental analysis of knockdown phenotype, study design and valuation. Elena V. Semina designed the study, supervised data collection, analysis and interpretation of results. All these authors participated in drafting the paper and all authors read and approved the final manuscript.

## Note added in proof

While this article was in revision, identification of the zebrafish *laminin alpha 1 *gene and associated notochord and blood vessel phenotype has been described by Pollard et al. [86].

## Note

**Table 1. Exon- intron boundaries of *laminin alpha 1*gene.**
